# GDNF-expressing STO feeder layer supports the long-term propagation of undifferentiated mouse spermatogonia with stem cell properties

**DOI:** 10.1038/srep36779

**Published:** 2016-11-09

**Authors:** Xiang Wei, Yuanyuan Jia, Yuanyuan Xue, Lei Geng, Min Wang, Lufan Li, Mei Wang, Xuemei Zhang, Xin Wu

**Affiliations:** 1 State Key Laboratory of Reproductive Medicine (SKLRM), Nanjing Medical University, Nanjing, Jiangsu 210029, China

## Abstract

The development of a stem cell culture system would expedite our understanding of the biology of tissue regeneration. Spermatogonial stem cell (SSC) is the foundation for lifelong male spermatogenesis and the SSC culture has been optimized continuously in recent years. However, there have been many inconveniences to reconstruct SSC self-renewal and proliferation *in vitro*, such as the frequent refreshment of recombinant cytokines, including GDNF, the essential growth factor for SSC maintenance. In the present study, we observed that both STO and MEF cells, which were previously used as feeders for SSC growth, did not express GDNF, but a GDNF-expressing STO feeder could support undifferentiated mouse spermatogonia propagation *in vitro* for three months without the refreshment of recombinant growth factor GDNF. The cell morphology, growth rate and SSC-associated gene expression remained identical to the SSCs cultured using previous methods. The transplantation of SSCs growing on these GDNF-expressing STO feeders could generate extensive colonies of spermatogenesis in recipient testes, functionally validating the stemness of these cells. Collectively, our data indicated that the further modification of feeder cells might facilitate the self-renewal and propagation of SSCs *in vitro*.

Tissue and organ regeneration mostly depends on stem cell self-renewal and differentiation capability. Spermatogonial stem cells (SSCs), comprising 1 in 3,000~4,000 cells of the adult mouse testes, are the only germline stem cells that support the extremely active spermatogenesis process^1^,^2^. SSCs can be harvested from donor testes and transferred into recipient testes to reestablish normal spermatogenesis^3^. Although a unique marker for SSCs is not available, the feasibility of a robust transplantation has provided functional validation for SSCs^3^. Similar to other types of stem cells, such as embryonic stem cells (ESCs), the development for SSCs culture *in vitro* would provide substantial value to study the mechanism of stem cell fate decision, their interactions with their niche microenvironment and their responses to a defined culture environment^4^. Knowledge of the mechanisms governing the fate decision of SSCs is essential for understanding spermatogenesis and the potential consequences of pathogenic insult.

Glial cell-derived neurotrophic factor (GDNF) is required for SSC survival, self-renewal and *in vitro* propagation^5^,^6^,^7^. Using human recombinant GDNF, two major *in vitro* culture systems for SSCs have been developed in the last decade. One is supported by a defined serum-free medium^6^ while the other is by a commercialized medium containing 1% fetal bovine serum^7^. Both systems require feeder layers either using STO (SIM mouse embryo-derived thioguanine and ouabain resistant fibroblast cell line) or MEF (Mouse embryo fibroblast cell line). In addition, a very frequent refreshment of several other recombinant growth factors, including FGF2, GFRA1 and/or LIF is required, which are extremely expensive and tedious. Meanwhile, there are significant differences of culture difficulty among variant strain backgrounds of mice^8^. Moreover, even though it has been shown that more than a dozen trophic factors, including GDNF, could influence SSC self-renewal or differentiation^9^, it remains unclear whether feeder cells can express and/or secrete enough GDNF or other factors to regulate the SSC fate decision *in vitro*.

In the present study, we aimed to investigate whether the expression of the growth factor GDNF in feeder cells is sufficient to support SSC propagation. By profiling the cytokine gene expression in STO and MEF cells, we found that none of the feeders expresses GDNF. We therefore generated a GDNF-expressing STO cell line by constructing lentivirus particles with mouse GDNF cDNA. Using this cell line as feeders, SSCs have grown and proliferated *in v*itro for over three months without apparent phenotype change or function loss. Our data suggest that the modification of feeder layers could be an alternative scheme to optimise the self-renewal and growth of mouse spermatogonial stem cells *in vitro.*

## Results

### Expression profile of trophic factor genes in STO and MEF cells

**Figure 1:**
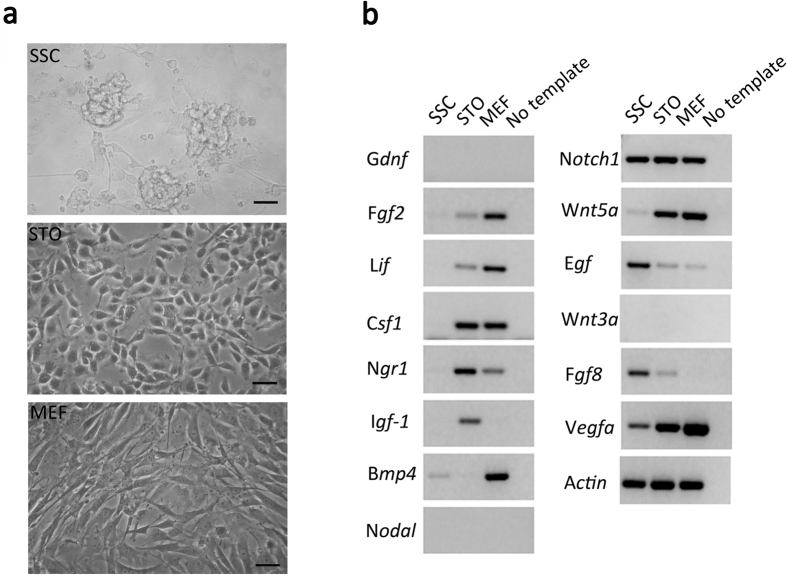
The expression profile of growth factor genes by feeder cells and SSCs. (**a**) The morphology of SSC clumps (upper panel), monolayer of STO cells (middle panel) and MEF cells (bottom panel) in culture dish, scale bar = 50 μm; (**b**) The semi-quantatitive PCR amplification for gene expression; mouse mRNA was extracted from SSCs, STO and MEF cells, and the β-*actin* gene served as a control. The experiments were repeated three times and the representative results were demonstrated in the figure.

Unlike SSCs, which grow with a typical clump-forming and grape shaping morphology, both the STO cells (distributed from Dr Alan Bradely) and MEF cells (used for ESC growth) in culture were shown as monolayer (Fig. 1a). Many trophic factors contributed to the self-renewal or fate decision of SSCs in testis niche or in *vitro*, therefore we first investigated whether the STO and MEF cells as well as the SSCs could express these genes by cDNA amplification. The identity of SSCs in culture was first verified by *Zbtb16*, *Lin28a*, *Dazl*, *Ddx4*, *Pou5f1* at mRNA levels and ZBTB16 and LIN28A at protein levels (Supplementary Fig. 1). Both STO and MEF cells have detectable expression of *Fgf2*, *Lif*, *Csf1*, *Ngr1*, *Notch1*, *Egf*, *Wnt5a* and *Vegfa*, while *Igf-1* expression was only detected in STO cells and *Bmp4* expression in MEF cells. Meanwhile, there was detectable expression of *Notch1*, *Egf*, *Fgf8* and *Vegfa* in SSCs, indicating the possibility of autocrine in SSCs. However, we are unable to detect the expression of neither *Gdnf*, nor *Nodal* or *Wnt3a* at any of the somatic feeder layer or stem cells themselves (Fig. 1b).

### Establishment of a GDNF-expressing STO line

**Figure 2:**
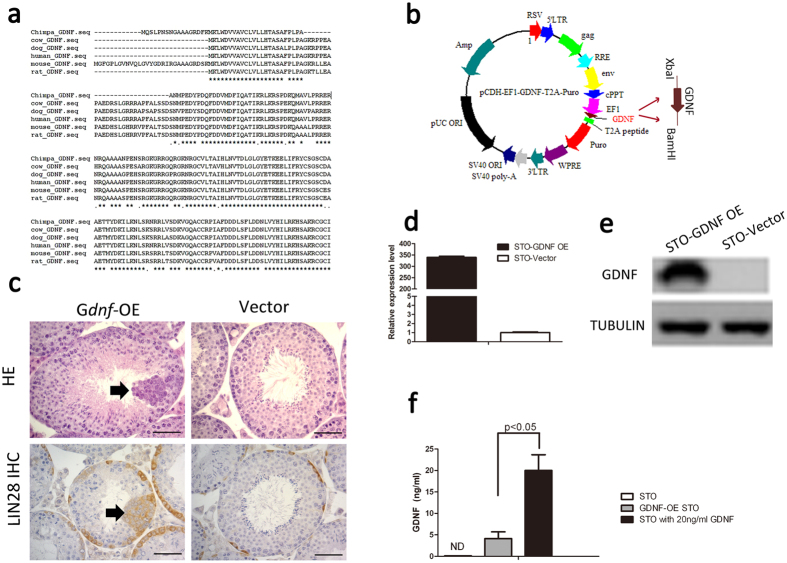
Lentivirus construction and STO cell transfection. (**a**) The amino acid sequence alignment for GDNF among mice, rats, humans, dogs, cows and chimpanzees; (**b**) The plasmid map of a 7095 bp of lentivirus plasmid used for packaging 723 bp mouse GDNF cDNA; (**c**) The histology and immunohistochemistry of LIN28A for recipient testes with GDNF lentivirus and vector virus transplantation; (**d**,**e**) quantatitive PCR and Western blot showing the high level of GDNF expression in GDNF-expressing STO cells; (**f**) The GDNF ELISA assay for the amount of detectable GDNF in the cultured medium, *p* < 0.05 (4 replicated wells in each group, ND indicated “not determined”), the error bars indicate the means ± SD.

All developed cultures for the SSCs derived from several species was supported by the NS0 cell (mouse myeloma cell)-derived human recombinant GDNF^6^,^7^,^10^,^11^. GDNF is a conserved protein among mice, rats, dogs, cows, chimpanzees and humans (Fig. 2a). To generate GDNF-expressing STO cells, we packaged the mouse GDNF cDNA into a pCDH-EF1-MCS-T2A-Puro vector plasmid and integrated a 7818bp sized lentivirus construct (Fig. 2b). We transplanted the lentivirus into six-week old C57BL/6 mouse testes. One month after transplantation, in the seminiferous tubules of the mouse testes, we found a significant proliferation and accumulation of LIN28A positive germ cells, which was used as a marker for spermatogonial progenitors^12^, demonstrating the biological function of over-expressing GDNF in the SSC niche by virus transplantation (Fig. 2c, HE and IHC staining). Using these lentivirus particles, we generated a STO cell line with a high level of GDNF expression, which are validated by a quantitative RT-PCR and Western Blot at the mRNA (Fig. 2d) and protein levels, respectively (Fig. 2e).

Next, we conducted an ELISA assay to measure GDNF concentration secreted from STO cells. As a result, we found that the average of 4.13 ± 1.56 ng/ml (approximately from 2.53 to 5.92 ng/ml) mouse GDNF was detected in the culture medium, which was generated by 200,000 culturing STO cells within 24 hrs (Fig. 2f and Supplementary datasheet 1).

### Long-term propagation of germ cells on a GDNF-secreting feeder layer

**Figure 3:**
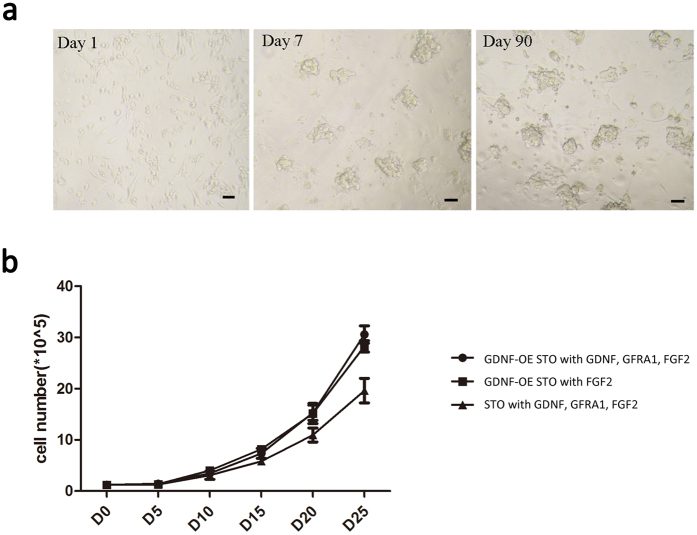
SSC propagation on a GDNF-secreting STO feeder layer. (**a**) A culture of a line of SSCs harvested from 6–8 days old C57BL/6 mouse testes. The morphology of SSC clumps formed at Day 1, 7 and Day 90. Scale bar = 50 μm; (**b**) The growth curve calculated from a line of SSCs (combined) supported by GDNF-expressing STO feeders with the addition of 20 ng/ml GDNF, 1 ng/ml FGF2 and 150 ng/ml GFRA1 (GDNF-OE STO with GDNF, GFRA1, FGF2), a line by GDNF-expressing STO feeders with 1ng/ml FGF2 (GDNF-OE STO with FGF2), and a line by normal STO feeders with GDNF, GFRA1, FGF2 (STO with GDNF, GFRA1, FGF2). The 25-day culture data were collected through three wells of each individual line of SSCs, the error bars indicate the means ± SD.

SSCs enriched germ cells were isolated from prepuberty mice of C57BL/6 or 129 × C57BL/6 ROSA^mT^/^mG^ tdTOMATO transgenic mice by CD90.2 conjugated magnetic bead sorting, and the cells were plated into culture wells with a pre-coated GDNF-expressing STO feeder layer, complying with previous methods^13^. In addition, 1ng/ml FGF2 was supplied into the medium for SSC culture, because we found the relative expression of FGF2 was much lower in STO when compared to Sertoli cells (Supplementary Fig. 2). We found that the initial clumps of SSCs could be easily established in culture (Fig. 3a, at day 1 and 7). A continued proliferation of SSCs was able to be achieved by a weekly subculture for three months *in vitro* (Fig. 3a, at day 90).

**Figure 4:**
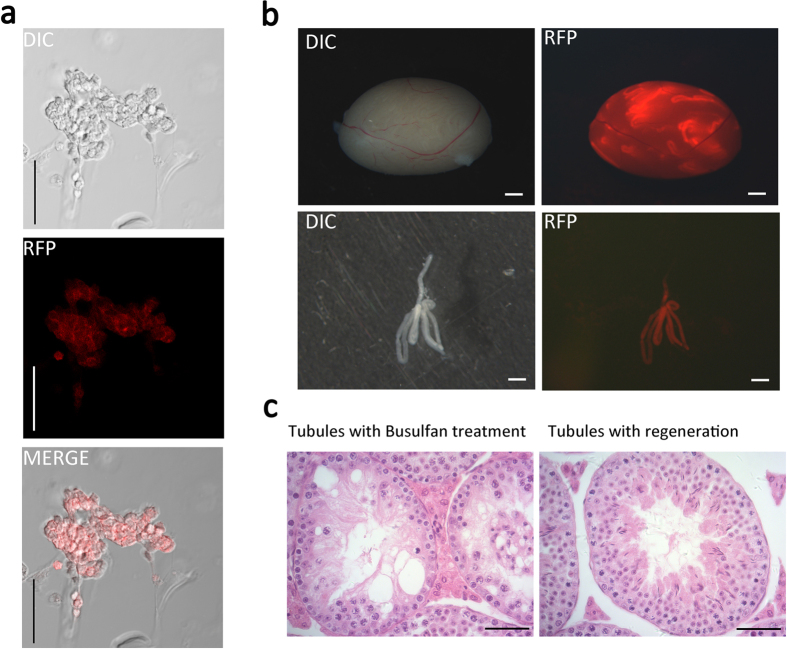
Mouse SSC transplantation. (**a**) A culture of testis SSCs harvested from 6–8 days old ROSA^mT/mG^(129 × C57BL/6 background) mice, a two-colour fluorescent Cre reporter allele allowing the strong and persistent expression of RFP driven by the ACTB promoter before Cre-induction. The SSC clumps therefore expressed cell membrane-localised red fluorescence. Scale bar = 50 μm; (**b**) Extensive colonies were observed by the transplantation of the ROSA^mT/mG^ mouse SSCs into 129 × C57BL/6 recipient mouse testes(upper panel), which is further visualised by regeneration fragments of spermatogenesis in the individual seminiferous tubule (lower panel). Scale bar = 1 mm; (**c**)Compared to the histology of paraffin sections from the busulfan-treated mouse testes, normal spermatogenic waves were found in the recipient testes after transplantation. Scale bar = 50 μm.

In total, we have established one line of SSCs from the mice with C57BL/6 background (Fig. 3a), and two lines of SSCs from ROSA^mT^/^mG^ transgenic mice with 129 × C57BL/6 background (Fig. 4a). In order to make a growth comparison of above SSC line (medium only supplied with 1ng/ml FGF2) with other SSC line supported by normal STO but medium supplied with recombinant GDNF, we then harvested SSCs from established lines and started with 100,000 above cells for each line to generate a 25-day proliferation comparison. As a result, we found no obvious alterations on the cell growth, the size and morphology of SSCs when compared with previous studies^6^,^7^ (Fig. 3b). The proliferation of these SSCs supported by GDNF-expressing STO feeder appeared even faster than cells supported by normal STO; however, adding additional recombinant GDNF to culture did not significantly improve the proliferation of SSCs on GDNF-expressing feeders (Fig. 3b).

### Functional validation of stemness for cultured germ cells

SSCs are capable of colonizing in recipient testes and maintaining long-term spermatogenesis, therefore spermatogonial cell transplantation provides a definitive method to define SSC presence and function in a given cell population. Using GDNF-expressing STO feeder layer, we have established a line of germ cells from ROSA^mT^/^mG^ tdTOMATO transgenic mice and these cell clumps are RFP fluorescence positive (Fig. 4a, DIC, RFP and Merged images). We transplanted the cultured germ cells into busulfan-treated mouse recipient testes. Three months later, we found many fluorescent-positive regeneration colonies of spermatogenesis in the recipient testes (Fig. 4b). Next, we dissected the testes and found, inside of seminiferous tubules, normal spermatogenic wave and elongated sperms which were not present in busulfan-treated tubules (Fig. 4c). These data, taken together, functionally validated the presence of stem cells in cultured germ cells.

### Phenotype analysis of cultured germ cells

**Figure 5:**
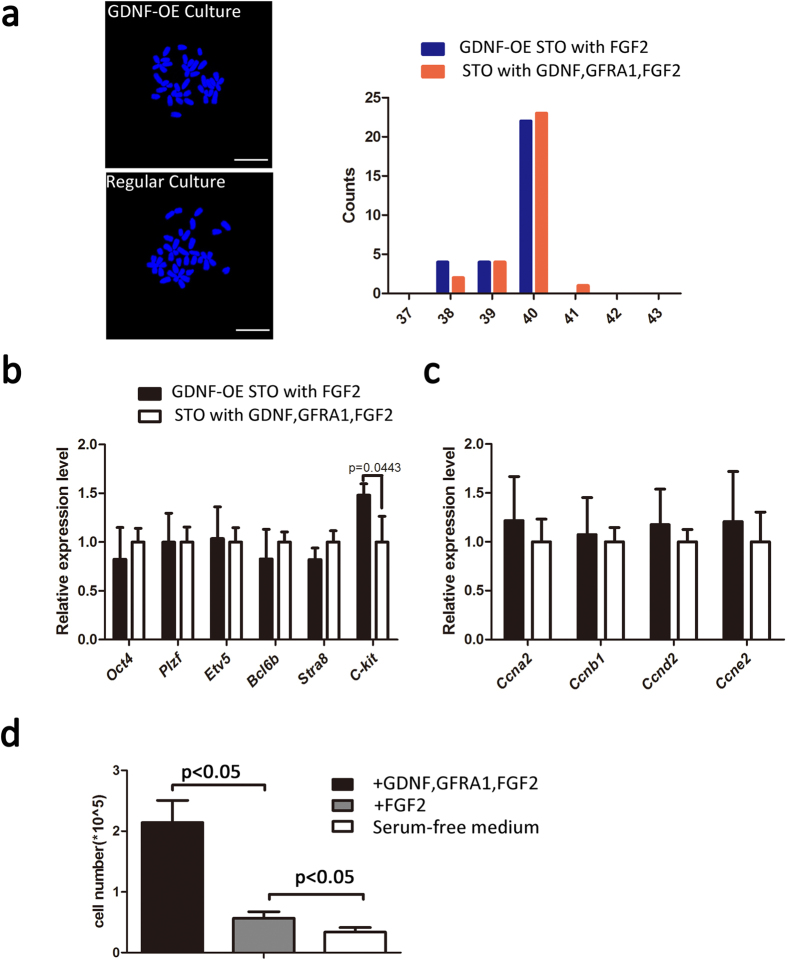
Karyotype analysis and SSC associated gene expression. (**a**) A metaphase spread and Karyotype analysis of SSCs cultured on GDNF-expressing STO feeders (GDNF-OE Culture) and normal STO feeder (Regular Culture). There was no obvious difference when based on a sum of thirty cells. Scale Bar = 10 μm; (**b**,**c**) quantitative PCR analysis of expression of self-renewal and differentiation associated genes and cell cycle associated genes, the error bars indicate the means ± SD; (**d**) The cell number for SSC growing for one week after the replacement of the GDNF-expressing STO feeders by normal STO feeder with or without growth factors, *p* < 0.05. From left to right, a culture with every-other-day refreshment of GDNF, FGF2, GFRA1, a culture with every-other-day refreshment of FGF2, and a culture without recombinant growth factors (serum-free medium only).

Furthermore, we did a karyotype analysis on cultured SSCs supported by GDNF-expressing STO feeders. Compared to previous culture methods with the frequent refreshment of exogenous GDNF, we observed no apparent loss on chromosome numbers when SSCs were cultured on GDNF-expressing feeders (Fig. 5a). We then investigated the expression of those genes that are functionally related to stem cell renewal (*Pou5f1*, *Zbtb16*, *Etv5*, *Bcl6b*), differentiation (*Stra8*, *C-kit*) (Fig. 5b), and cell cycling (*Ccna2*, *Ccnb1*, *Ccnd2*, *Ccne2*) (Fig. 5c). We did not detect any significant difference in gene expression related to cell renewal and cycling. Although the *C-kit* gene was statistically higher (p = 0.0443, t-test) for SSCs cultured on GDNF-expressing STO feeders than for the control culture (STO with GDNF, FGF2,GFRA1, Fig. 5b), the expression of both *Stra8* and *C-kit* remained at fairly low levels in the former culture, indicating the majority of germ cells kept undifferentiated in the GDNF-secreting STO culture. We also found no changes for other SSC-associated genes, including *Gfra1*, *Sall4*, *Vasa*, *Id4*, *Pax7* (Supplementary Fig. 3). These data indicated that SSC culture supported by GDNF-expressing feeders was likely identical to previously reported SSC culture.

*In vitro* proliferation of SSCs was dependent on GDNF. In order to determine the response to GDNF depletion for these SSCs who grown on the GDNF-expressing STO, we then collected these cells and plated them on the normal STO feeder without GDNF expression. One week proliferation was compared by omitting recombinant GDNF or FGF2 from the serum-free medium (Fig. 5d). Unsurprisingly, we found these SSCs experienced a rapid loss in the culture by GDNF withdrawal; however, the daily refreshment of FGF2 was beneficial to SSC survival and maintenance (Fig. 5d*, p* < 0.05).

## Discussion

Spermatogonial stem cells (SSCs) are the foundation of spermatogenesis throughout adult life. The development of techniques to maintain SSCs *in vitro* essentially allows for improving our understanding of the processes and regulation of spermatogenesis and aids in solving problems associated with male infertility^14^,^15^. Several mammalian germ cell lines have been previously constructed through the over-expression of genes, such as the simian virus 40 large tumor antigen, temperature-sensitive mutant of p53, and mouse telomerase^16^,^17^; however, it is only by supplementing the growth factor GDNF, a member of the TGFβ family into the testis cell culture, so that a successful establishment of stem cell lines for(from) germline lineages with the capability of long-term self-renewal and complete differentiation through functional transplantations^6^,^7^, is observed.

Although recent data showed that feeder-free culture could support germ cell proliferation with a lower SSC frequency after transplantation^18^, both STO and MEF cells derived from mouse embryo are beneficial to mouse SSC growth, which is similar to the cultures of ESCs and/or PGCs (primordial germ cells)^19^,^20^,^21^. It was speculated that growth factors and cytokines secreted by the feeders and interaction between stem cells and feeder cells can be attributed to beneficial effects to stem cells^9^. Many growth factors and cytokines have been reported to regulate SSC survival and proliferation^9^ and our data indicated that both STO and MEF cells have detectable expression of growth factor genes including *Fgf2*, *Lif*, *Csf1* and *Egf*. Of those factors, endogenously produced FGF2 has also been shown to be beneficial for mouse SSCs, which is likely through MAP2K1 kinase activation to regulate downstream genes^22^,^23^ and a recent study showed that FGF2 even independently supports *in vitro* SSC expansion without GDNF^24^. Furthermore, the growth factors LIF and BMP4 could balance self-renewal and differentiation in mouse SSCs and BMP4 could induce differentiation in Kit-negative spermatogonia through its Alk3 and Smad5 receptor signaling pathways^25^. We found that *Bmp4* was only expressed by MEF feeder cells, but a direct culture comparison of SSC culture on STO and MEF cells and an evaluation of the potential effects of BMP4 secreted by feeders have not been reported. Nevertheless, both STO and MEF cells did not express GDNF. It was shown that the GDNF released from testis Sertoli cells and the SSCs population in the cryptorchid testes expanded by approximately 20 fold^26^ when human GDNF cDNA was transfected into mouse Sertoli cells by an *in vivo* transplantation. Testis Sertoli cell is the critical somatic cell lineages mediated SSC fate *in vivo*. Although most of the Sertoli lines established either by transgenic animals or gene infection had been widely applied for spermatogenesis mediation or investigation of Sertoli cells themselves^27^,^28^,^29^, the application of Sertoli cells for SSC growth has not been validated. Recently testosterone-regulated GDNF secretion by peritubular myoid cells has also been observed, which ensured the optimal GDNF level for repopulating the undifferentiated spermatogonia including SSCs^30^. However, testis somatic cell feeders were likely to inhibit SSCs survival and replication *in vitro*^9^, and it is unknown whether the overexpression of GDNF and/or other growth factors in STO or MEF could support SSC survival or the long-term proliferation of a SSC line.

Taking this information into consideration, in the present study we generated a GDNF-secreting STO cell line and applied it as a feeder layer to support SSCs *in vitro*. The concentration of mouse GDNF is about 4.13ng/ml generated by 200,000 STO cells within one day, which is about 25% of the dose applied in previous culture by human recombinant GDNF (20 ng/ml), but every other day refreshment is required for previous culture. Even though, we found no obvious defects on the size and morphology of SSC clumps. Significantly, we found a better growth rate than culture used in previous studies (Fig. 3b). The results strongly suggested that a direct and durative secret of GDNF from feeder layers might be more beneficial to SSC growth than every other day refreshment of human recombinant factors into medium.

Collectively, we found that a successful SSC propagation for over three months was achieved on GDNF-expressing STO feeders. Our results strongly indicated that the modification of a feeder layer could be useful to optimise the culture system for SSCs and avoid the tedious work and high costs that accompany the addition of recombinant growth factors to culture.

## Materials and Methods

### Cell culture and donor mice

The STO cells (Passage 4 to 6) were gifted by Dr. Ralph Brinster from the University of Pennsylvania, USA with the permission of Dr. Alan Bradley from the Wellcome Trust Sanger Institute, Cambridge, UK. The STO cells were cultured in DMEM medium (Life Technology) supplemented with 7% foetal bovine serum (FBS, Hyclone). Before applying for the SSC culture, the cells were inactivated with 10
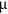
g Mitomycin-C (MITC) ( Sigma-Aldrich) following the previous protocol^13^. An initial purification of SSC-enriched germ cells was achieved through cell sorting according to the previous protocol^13^. Briefly, testes were harvested from of 6–8 days old C57BL/6 background mice or 129 × C57BL/6 background ROSA^mT^/^mG^ tandem dimer^13^ transgenic mice (The Jackson Laboratory, Maine, USA). After the digestion with 1 mg/ml Collagenase IV (Sigma-Aldrich), 0.25% Trypsin-EDTA (Life Technology) and 7 mg/ml DNase (Sigma-Aldrich), single cell suspension was incubated with CD90.2 conjugated microbeads, and applied with a magnetic-activated cell sorting process. Approximately 100,000 CD90.2 positive cells were seeded on MITC inactivated STO cell feeders. Both normal STO and GDNF-expressing STO cells were plated at 100,000 cell per well on 24-well plates. A serum-free medium containing 1 ng/ml FGF2 (BD Biosciences) was used as the culture as described in the previous protocol^13^. A synchronous operation for medium changes was maintained for either cells cultured on normal or on GDNF-expressing STO feeders, while the former also required 20 ng /ml of the recombinant growth factor GDNF(R&D Systems) and 150 ng/ml of GFRA1(R&D Systems). The SSCs were subcultured to new STO feeders every five days at a ratio of 1:2 or 1:3.

All the procedures for animal studies were approved by the Institutional Animal Care and Use Committee (IACUC) of Nanjing Medical University (ID: 2011082112). In addition, all experiments were performed in accordance with the institutional guidelines of Nanjing Medical University.

### Plasmid construct and transfection

The mouse CDS sequence of GDNF was taken from the GeneBank database. We amplified the GDNF sequence from the C57BL/6 mouse strain testis cDNA and constructed GDNF cDNA into the pCDH-EF1-MCS-T2A-Puro vector by performing a restriction enzyme cutting with XbaI and BamHI. A CaCl_2_-mediated plasmid transfection was administered using the protocol provided by System Biosciences (pCDH cDNA cloning and Expression lentivectors). Human HEK293T cells were used to generate the lentivirus suspension. The virus was collected from a 10 cm culture dish when HEK293T cell density was approximately 80%. The virus suspension was diluted (1:1) with a STO culture medium, and 100,000 STO cells were placed in each well of a six-well plate and incubated with the virus for 24 hours; then, the fresh STO medium was supplied and the cells were eventually harvested for verification within 3 days.

### Recipient mice and SSC transplantation

129/SvCP × C57BL/6 heterozygous male mice (Jackson Laboratory) were prepared as the recipient mice. 55 mg/kg Busulfan (Sigma-Aldrich) were administered to mice at 2 months old, and the recipients were then ready for transplantation five weeks after. For the lentiviral transduction of testis tissue, the GDNF lentivirus particles were concentrated by 100-fold by ultracentrifugation at 50,000 g for 90 min at 4 °C (Optima L-100 XP Ultracentrifuge), and a 10 μl concentrated lentivirus medium was injected into the rete of each testis of the 6-week-old C57BL/6 (B6) mice. For cell transplantation, 100,000 SSCs (ROSA^mT^/^mG^ tdTomato) cultured on the GDNF-expressing STO layer were transplanted into the rete of each testis of the Busulfan- treated recipient mice. Three months after transplantation, the testes were harvested and the donor-cell derived colonies were visualised by a fluorescence dissecting microscope (Zeiss Lumar V12).

### RNA isolation and real-time PCR

The total RNA was isolated by Trizol Reagent (Life Technology), followed by a chloroform and isopropanol separation. First-strand cDNA was synthesised with a reverse transcription kit (Takara Bio). A quantatitive PCR was performed with the SYBR Premix Ex Taq II mixture (Takara Bio) in the 20μl reaction system by StepOne Plus system (Applied Biosystems). The primer information is listed in the supplementary information. The expression level of each gene was calculated by a 2^−ΔCT^ value.

### ELISA Assay

Starting with approximately 200,000 cells per well in a 12-well culture plate, normal STO and GDNF-expressing STO cells were cultured in 1 ml STO medium for 24 hrs. Medium were immediately collected and applied for the ELISA assay. The procedure was followed by an instruction in a commercial GDNF mouse ELISA Kit (Cat#ab171178, mouse GDNF ELISA kit, Abcam). OD (optical density) values were generated through the Microplate Reader (BioTek Synergy2, USA). 20 ng/ml human recombinant GDNF in STO medium served as positive control.

### Karyotyping analysis

The Cultured SSCs were incubated with 1 mg/ml of Colchicine (Dahui BioCompany, China) before the trypsin digestion. The Cells were then incubated with 75 mM potassium chloride for 15 min and fixed with methanol/acetic acid (3:1) for 1 h. A metaphase spread was performed and the sample slides were stained with DAPI (Sigma-Aldrich) and analysed by confocal microscopy (LSM 700, Zeiss).

### Histology and IHC

The testis tissue was first fixed with Hartman solution (Sigma-Aldrich) for 24 h, then embedded in Paraffin (Leica) and sectioned by 5 μm. After an overnight incubation at 65 °C, the section slides were de-paraffinised and treated with 3% hydrogen peroxide(Sigma-Aldrich). Primary antibodies against LIN28A (1:5000, Abcam) was used to visualise the undifferentiated spermatogonia in testes. Hematoxylin-eosin (Sigma-Aldrich) staining was applied for the histology of the recipient mouse testes.

### Statistics

All data are presented as means ± SD. Statistic data were analyzed by t-test.

## Additional Information

**How to cite this article**: Wei, X. *et al.* GDNF-expressing STO feeder layer supports the long-term propagation of undifferentiated mouse spermatogonia with stem cell properties. *Sci. Rep.* **6**, 36779; doi: 10.1038/srep36779 (2016).

**Publisher’s note:** Springer Nature remains neutral with regard to jurisdictional claims in published maps and institutional affiliations.

## Supplementary Material

Supplementary Information

Supplementary File 1

Supplementary File 2
