# Supplementary material for: GDNF-expressing STO feeder layer supports the long-term propagation of undifferentiated mouse spermatogonia with stem cell properties
Source: Sci Rep. 2016 Nov 9;6:36779. doi: 10.1038/srep36779 (PMC5101510; doi:10.1038/srep36779)
Supplement: Supplementary Information [file srep36779-s1.doc]

**Title:** GDNF-expressing STO feeder layer supports the long-term propagation of undifferentiated mouse spermatogonia with stem cell properties

Xiang Wei^1^, Yuanyuan Jia^1^, Yuanyuan Xue^1^, Lei Geng, Min Wang, Lufan Li, Mei Wang, Xuemei Zhang, Xin Wu

Supplemental figures 1 Characterization of SSCs in culture.


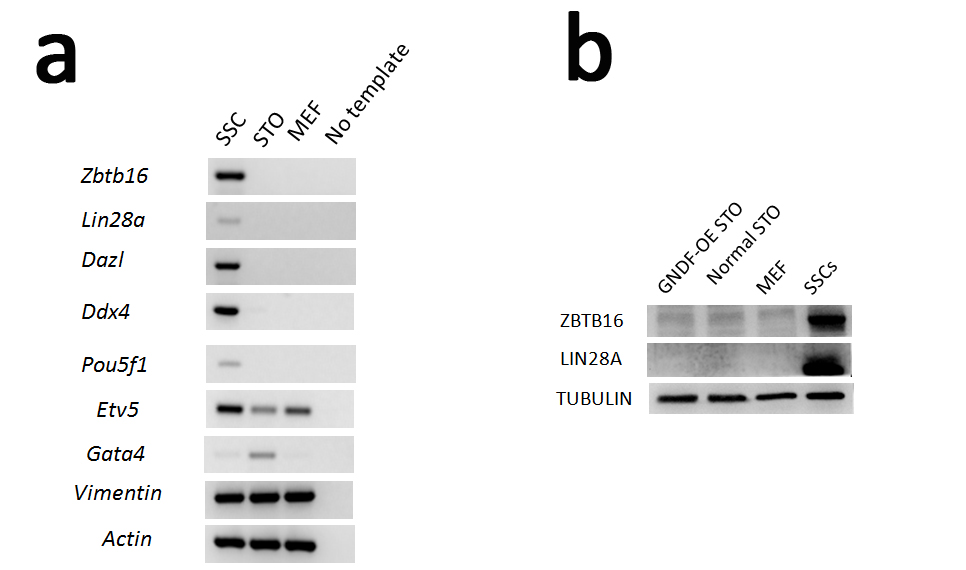


1. The semi-quantatitive PCR to verify SSC associated gene expression in SSCs; (b) The western Blot of ZBTB16 and LIN28A in SSCs, STO and MEF cells.

Supplementary figure 2 The relative expression of fgf2.


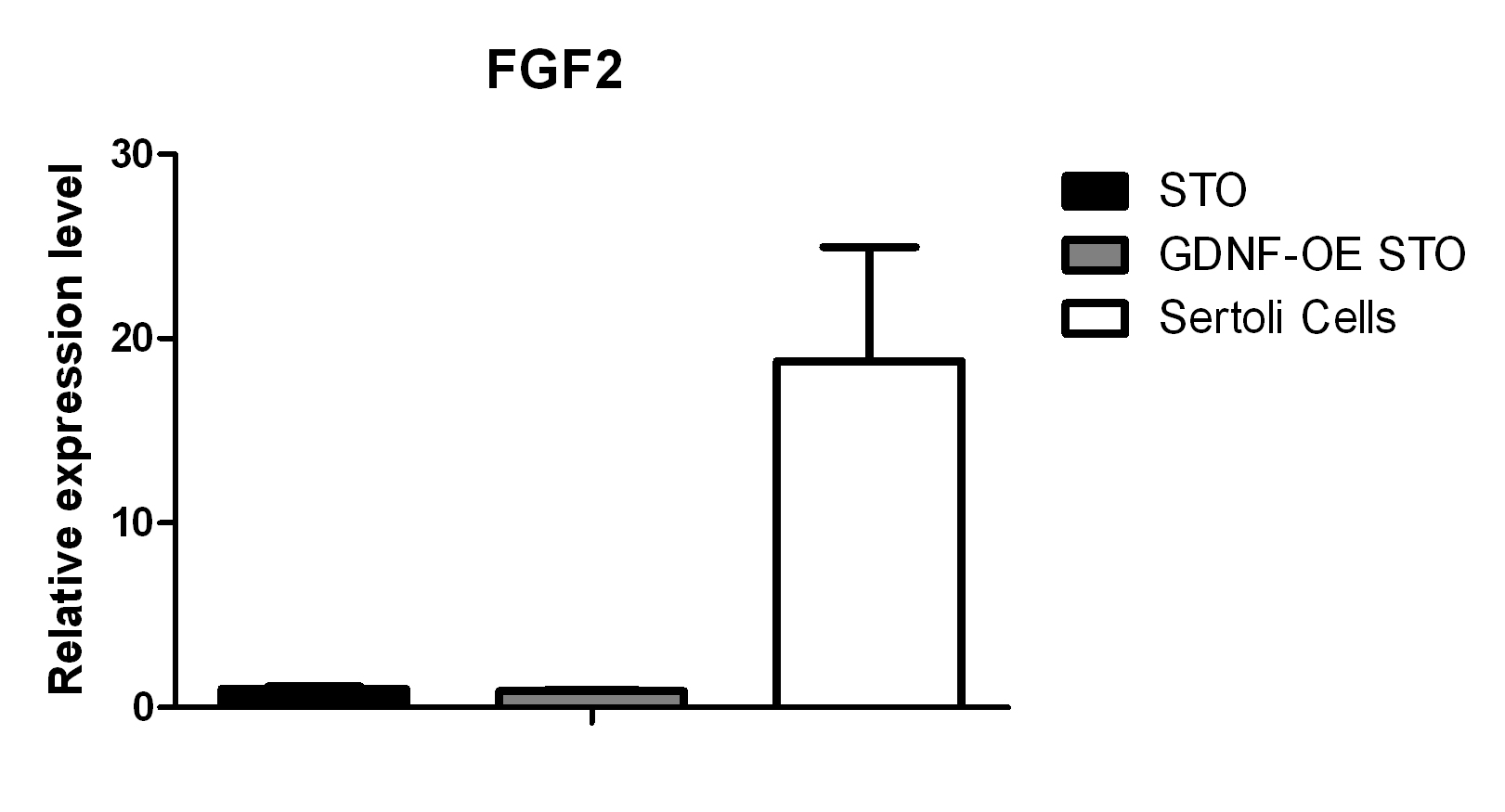


The FGF2 expression in normal STO (black), GDNF-OE STO Cells(gray), and primary isolated Sertoli Cells(white). The error bars indicate the means±SD.

Supplementary figure 3 Genes expression associated to SSCs.


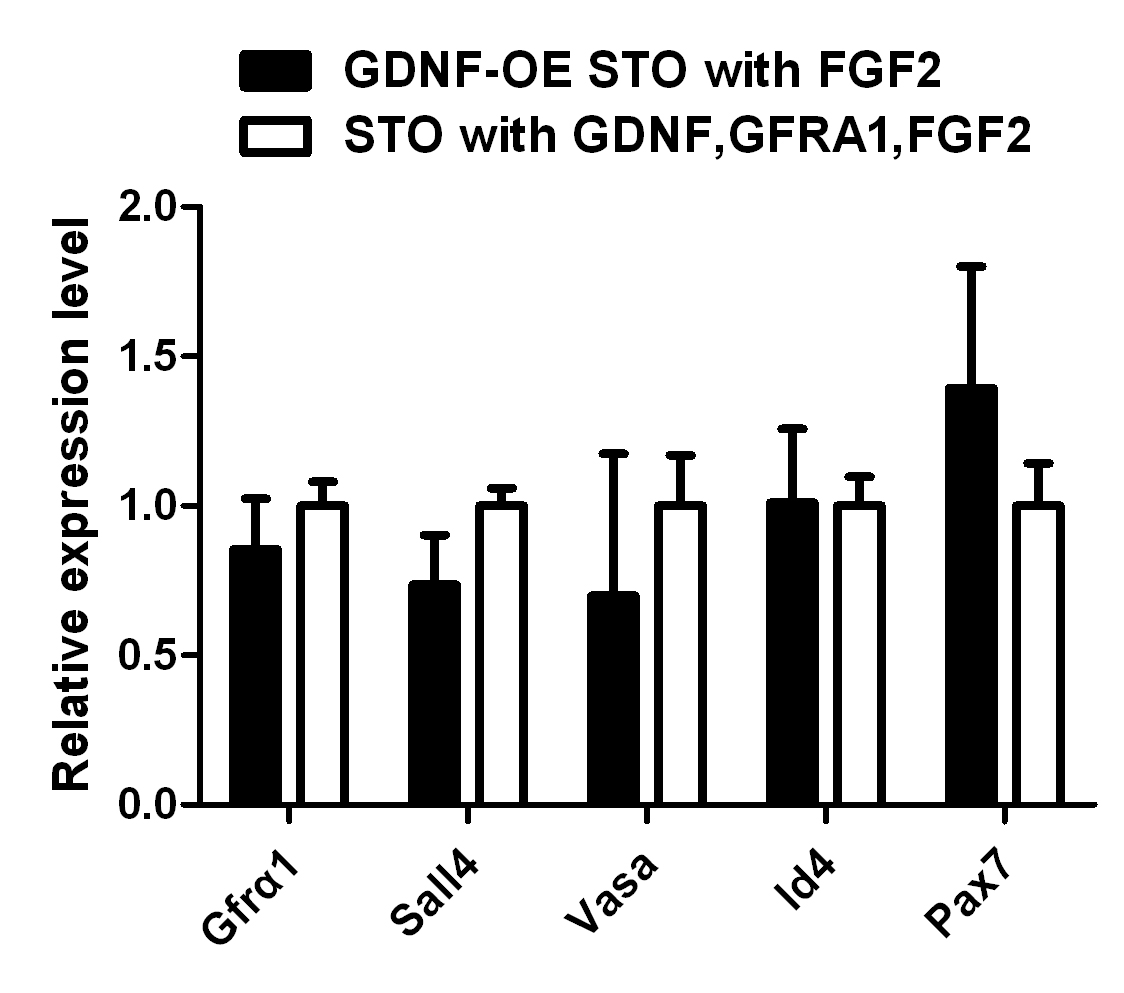


The quantitative PCR analysis of expression of genes associated to SSCs supported by GDNF-expressing STO (GDNF-OE Culture, black) or normal STO (Regular Culture, white). The error bars indicate the means±SD
