# Supplementary material for: GDNF-expressing STO feeder layer supports the long-term propagation of undifferentiated mouse spermatogonia with stem cell properties
Source: Sci Rep. 2016 Nov 9;6:36779. doi: 10.1038/srep36779 (PMC5101510; doi:10.1038/srep36779)
Supplement: Supplementary File 1 [file srep36779-s2.doc]

**Supplementary information**

**Title:** GDNF-expressing STO feeder layer supports the long-term propagation of undifferentiated mouse spermatogonia with stem cell properties

Xiang Wei^1^, Yuanyuan Jia^1^, Yuanyuan Xue^1^, Lei Geng, Min Wang, Lufan Li, Mei Wang, Xuemei Zhang, Xin Wu

**Primer information**

| **PCR primer for data in figure 1** | **Forward** | **Reverse** |
| --- | --- | --- |
| *Gdnf* | AAGTTATGGGATGTCGTGGC | CTGGTCAGGATAATCTTCAGGC |
| *Nrg1* | ATGGAGATTTATCCCCCAGACA | GTTGAGGCACCCTCTGAGAC |
| *Csf1* | GCTGCCCTTCTTCGACATG | CCTTCAGGTGTCCATTCCCA |
| *Wnt3a* | ATCTTTGGCCCTGTTCTGGA | CAATGTCCTCACTACAGCCG |
| *Fgf8* | CAGAAGACGGAGACCCCTT | CCGTGTAGTTGTTCTCCAGC |
| *Nodal* | ACATGTTGAGCCTCTACCGA | ACCAGATCCTCTTCTTGGCT |
| *Lif* | CCTCTTCCCATCACCCCTGTAAAT | ACTTGGTCTTCTCTGTCCCGTTGC |
| *Wnt5a* | TCCTATGAGAGCGCACGCAT | CAGCTTGCCCCGGCTGTTGA |
| *Igf-1* | AAGGCAGTTTACCCAGGCTC | TCTTTATTGCAGGTGCGGTCA |
| *Bmp4* | TTTGGCCATGATGGCCGGGGCCATACCTT | TCAGCGGCATCCACACCCCTCTACCACCAT |
| *Vegfa* | GAGCTCATGGACGGGTGAG | CTGGGACCACTTGGCATGG |
| *Egf* | TTTGACAAGTGGCAGGAGGTC | CAGGCGATGAACAACCAGTG |
| *Notch1* | ACAGTGCAACCCCCTGTATG | CCGCAGAAAGTGGAAGGAGT |
| *Fgf2* | GGCTGCTGGCTTCTAAGTGT | GTCCCGTTTTGGATCCGAGT |

| **PCR primer for data in figure5 & supplementary figure 2** | **Forward** | **Reverse** |
| --- | --- | --- |
| *kit* | TGTGGCTAAAGATGAACCCTC | ACACTCCAGAATCGTCAACTC |
| *Stra8* | TTAAACCAGGAACCAGAGC | AAGACAAGGCAGTATAACTCTAGC |
| *Etv5* | CACCATGTATCGAGAGGGGC | GAGCAACCTCTTCCGGTTCT |
| *Bcl6b* | CGGAGCACGTTTTAACCGAC | CAGGGGTCACAGTGGTATGG |
| *Zbtb16* | CCCTATGAGTGTAATGGCTGTG | TTCTCAGGTGCTTGATCATGG |
| *Pou5f1* | TAGGTGAGCCGTCTTTCCAC | GCTTAGCCAGGTTCGAGGAT |
| *Ccna2* | GCTCAAGACTCGACGGGTTGC | GCTGCATTAAAAGCCAGGGCATC |
| *Ccnb1* | TCCCTCGGTGGGATTCAAGTGC | CAGGAGTGGCGCCTTGGTATGG |
| *Ccnd2* | CTGCGGAAAAGCTGTGCATT | AACTTGAAGTCGGTAGCGCA |
| *Ccne2* | GACGTTCATCCAGATAGCTCAG | AAAAGGCACCATCCAGTCTAC |
| *Actin* | CCGTAAAGACCTCTATGCC | CTCAGT AACAGTCCGCCTA |
| *Gfrα1* | CTCGGAATCCAGCCTACGTC | CACTTGTCCTCTCGTGTGCT |
| *Sall4* | CCCTGGGAACTGCGATGAAG | TCAGAGAGACTAAAGAACTCGGC |
| *Vasa* | TGATTCAGGCAATGGTGACACT | TTCCCAGACCCTGTTTGAGC |
| *Id4* | CGTTATCGACTACATCCTGGAC | TCTTAATTTCTGCTCTGGCCC |
| *Pax7* | TCAAGCCAGGAGACAGCTTG | TAGGCTTGTCCCGTTTCCAC |

| **CDS-primer** | **Forward** | **Reverse** |
| --- | --- | --- |
| *Gdnf* | GCTCTAGAGCCACCATGAAGTTATGGGATGTC | CGGGATCCGATACATCCACACCGTTTAG |
